# Genomic Characterization of Crossbred-Driven Adaptation in the Endangered Yangba Cattle of China

**DOI:** 10.3390/ani15071065

**Published:** 2025-04-06

**Authors:** Bao Cai, Yandong Kang, Ziqiang Ding, Shaoke Guo, Mengli Cao, Liyan Hu, Ben Zhang, Xingdong Wang, Jie Pei, Qianyun Ge, Lin Xiong, Xiaoyun Wu, Xian Guo

**Affiliations:** 1 Key Laboratory of Yak Breeding of Gansu Province, Lanzhou Institute of Husbandry and Pharmaceutical Sciences, Chinese Academy of Agricultural Sciences, Lanzhou 730050, China; 82101235499@caas.cn (B.C.); kangyandong0901@163.com (Y.K.); dingziqiang1997@163.com (Z.D.); gsk1125@163.com (S.G.); caomengliaaa@163.com (M.C.); huliyan2020@163.com (L.H.); zhangbencaas@163.com (B.Z.); wxd17339929758@163.com (X.W.); peijie@caas.cn (J.P.); geqianyun@caas.cn (Q.G.); xionglin@caas.cn (L.X.); 2 Key Laboratory of Animal Genetics and Breeding on Tibetan Plateau, Ministry of Agriculture and Rural Affairs, Lanzhou 730050, China

**Keywords:** endangered Yangba cattle, whole-genome resequencing, phylogeny, population genetics, gene introgression

## Abstract

This study unveils the unique origins, evolution, and genetic variations of the Yangba cattle, an endangered breed in China, through a comparative genomic analysis involving 202 individuals from 21 domestic and international breeds. Genetic component analysis revealed that the Yangba cattle comprise four ancestral lineages: Eurasian taurine (18%), East Asian taurine (26%), Chinese indicine (39%), and Indian indicine (17%). Their high genetic diversity and low inbreeding coefficient set them apart significantly from mainstream commercial breeds. Gene introgression analysis indicated that the influx of genetic material from East Asian taurine has enhanced the Yangba cattle’s adaptability to environmental stress, while the introgression from Chinese indicine has endowed them with unique advantages in muscle development and tissue repair. A genome-wide selection scan identified strong positive selection signals for genes such as *ABCC2*, which is involved in immune regulation, and *NCOA3*, which plays a role in growth regulation, in the Yangba cattle. This study systematically elucidates, for the first time, the composite ancestral composition and mechanisms of adaptive evolution in Yangba cattle. These findings offer critical insights into the conservation and sustainable utilization of endangered cattle resources and underscore the importance of implementing effective breeding programs.

## Simple Summary

Local indigenous livestock breeds are characterized by their rich genetic diversity and a long history of selective breeding, granting them notable advantages in environmental adaptability, forage tolerance, and disease resistance. However, the rapid advancement of modern animal husbandry has shifted the primary focus towards economic efficiency. As a result, many local breeds find themselves at a disadvantage in the competitive landscape of contemporary commercial practices due to their comparatively lower productivity and less substantial economic returns. The Yangba cattle, an endangered breed native to China, exemplify this challenge, lacking clear documentation on their origins, evolutionary history, and adaptive mechanisms. To address these gaps, we undertook a comprehensive comparative genomic analysis involving 202 individuals from 21 domestic and international breeds. Our objective was to uncover the unique genetic origins, evolutionary pathways, and distinctive variations that define the Yangba cattle, thereby enriching our understanding of this remarkable breed.

## 1. Introduction

The origin of domestic cattle in China can be traced back to the Middle East, entering China from the eastern and northern regions around 3900 BC and spreading into the Central Plains [1]. Depending on their lineage sources, Chinese cattle can be categorized into three types: taurine cattle (*Bos taurus*), indicine cattle (*Bos indicus*), and hybrids of both, with the proportion of zebu ancestry showing a gradual decline from south to north [2]. Based on geographical areas, Chinese domestic cattle are divided into Northern cattle, Central Plains cattle, and Southern cattle [3]. Through long-term genetic evolution, Chinese domestic cattle have gradually formed various subspecies and breeds adapted to the different climates and terrains across China. Local indigenous breeds are characterized by rich genetic diversity and a long history of selective breeding, providing significant advantages in environmental adaptability, forage tolerance, and disease resistance [3,4,5]. However, with the rapid development of modern animal husbandry, livestock production has increasingly focused on economic benefits. Many local breeds are at a disadvantage in the competition of modern commercial animal husbandry due to lower productivity and less notable economic benefits [6,7]. Additionally, environmental degradation, habitat loss, and the introduction of foreign breeds have led to a sharp decline in the population numbers of some local cattle breeds (such as Zhoushan cattle [8], German Black Pied cattle [9], Swedish cattle [10], etc.), pushing them to the brink of extinction.

Yangba cattle are a distinctive local breed in China, known for their strong adaptability and growth potential. They are an integral part of local agricultural production, ecological protection, and sustainable development. Primarily distributed in Yangba and the surrounding townships of Kang County, Longnan City, Gansu Province, Yangba cattle are characterized by their straight back and waist, have strong hill-climbing abilities, and are suitable for grazing on mountainous and forested slopes. In the past, Yangba cattle were one of the primary labor resources locally, widely used for plowing and mountain work. However, with the transformation of socio-economic models and the continuous improvement of mechanization levels, the number of Yangba cattle being raised has decreased annually, facing the crisis of extinction. The disappearance of any local cattle breed would lead to the permanent loss of its unique gene pool, which may contain special genes needed for the future, such as disease resistance, stress tolerance, or high productivity genes [10,11]. This loss not only poses a threat to the development of animal husbandry but also brings immeasurable impacts on the conservation and sustainable use of global genetic resources. Yangba cattle play an important role not only in ecological protection but also have unique significance in maintaining the agricultural ecological balance and biodiversity of the local area. Their ability to adapt to harsh environments and their suitability for mountain ecology make them irreplaceable in maintaining the stability of mountain ecosystems. Therefore, protecting Yangba cattle is not only necessary for maintaining biodiversity but also critical for ensuring the sustainable development of future animal husbandry. Despite being located in the border area of the Central Plains, research on the origin and evolution of Yangba cattle remains inadequate, with extremely scarce records and data. Especially concerning their genetic diversity and adaptive mechanisms at the genetic level, there has been insufficient exploration, posing significant challenges for the protection and rational utilization of this breed.

In recent years, with the rapid development of genomics technology, whole-genome sequencing and analysis have provided powerful tools for studying the genetic diversity, origin, evolution, and functional gene discovery of local breeds [12,13]. Through whole-genome studies of local cattle breeds, it is possible to reveal their unique genetic variations, decipher the genetic basis of their adaptability and economic traits, and provide scientific evidence for breed conservation, genetic improvement, and sustainable utilization [14,15]. Therefore, this study aims to compare the genetic variation information of the endangered Yangba cattle with other domestic and international breeds. By analyzing at the whole-genome level, we aim to uncover the unique genetic variations of Yangba cattle, explore their origin, evolution, and genetic diversity, and identify the main genetic characteristics responsible for their desirable traits. This will provide a theoretical reference for the conservation of endangered breeds and the scientific utilization of breed resources.

## 2. Materials and Methods

### 2.1. Ethics Statement

All experimental procedures involving cattle were conducted in strict compliance with the Regulations for the Administration of Experimental Animals (approved by the State Council of the People’s Republic of China). Written informed consent was obtained from all cattle owners prior to sample collection. This study was formally approved by the Animal Administration and Ethics Committee of Lanzhou Institute of Husbandry and Pharmaceutical Sciences, Chinese Academy of Agricultural Sciences (CAAS) (Permit No.: SYXK-2024-0023). Additionally, the study adheres to the guidelines outlined in the ARRIVE Checklist to ensure transparency and rigor in reporting. All protocols, including sample collection procedures, were executed with explicit authorization from the animal owners and under the supervision of institutional ethics standards.

### 2.2. Sample Collection and Whole-Genome Resequencing

To investigate the whole-genome selection characteristics of Yangba cattle, we conducted venous blood collection from 20 individuals (12 bulls and 8 cows) across several areas including Baiyang Town, Lianghe Town, Yangba Town, Tongqian Town, and Sanhe Town in Kang County, Longnan City. The collected blood samples were promptly preserved at −80 °C. Considering the endangered status of the Yangba cattle, characterized by a limited number of existing individuals, we expanded our sampling range by randomly selecting additional samples from various townships within their primary distribution areas to ensure representativeness. Genomic DNA was extracted using the standard phenol–chloroform method. The samples were then sent to Kangsheng Xuyuan Biotechnology (Wuhan, China) Co., Ltd. for whole-genome resequencing. For comparative genomic analysis, we also collected 182 additional samples, comprising 106 samples from domestic breeds and 76 samples from foreign breeds (Supplementary Tables S1 and S2).

### 2.3. Data Quality Control and Alignment

To ensure the quality of the sequencing results, we used the fastp software (v0.24.0) to perform quality control on the obtained reads, filtering out low-quality sequences [16]. The processed sequencing reads were then aligned to the cattle reference genome (NCBI version: ARS-UCD2.0) using the BWA software (v0.7.18) [17]. After alignment, the BAM files were sorted using the Samtools software (v1.10) to generate sorted BAM files. The Sambamba software (v1.0.1) was subsequently employed to identify and remove PCR-induced duplicate reads from the aforementioned BAM files [17,18,19].

### 2.4. Single Nucleotide Polymorphism (SNP) Detection and Annotation

For SNP detection, we utilized the HaplotypeCaller, GenotypeGVCFs, and SelectVariants modules from GATK (v4.3.0.0). To ensure high-quality SNPs, we applied the VariantFiltration module in GATK with the following threshold values: Quality by Depth (QD) < 2.0, Quality Score (QUAL) < 30.0, Strand Odds Ratio (SOR) > 3.0, Fisher Strand Bias (FS) > 60.0, Mapping Quality (MQ) < 40.0, MQ Rank Sum Test < −12.5, and Read Position Rank Sum Test < −8.0 [20]. Using BCFtools (v1.10.2), we retained SNP sites with a genotype missing rate not exceeding 20% (F_MISSING < 0.1) and a minor allele count greater than 2 (MAC > 2) [21]. Each breed’s SNPs were annotated using ANNOVAR, referencing the annotation file for the Bos taurus reference genome (available at: https://ftp.ncbi.nlm.nih.gov/genomes/all/GCF/002/263/795/GCF002263795.3ARS-UCD2.0/GCF002263795.3ARS-UCD2.0genomic.gff.gz) (accessed on 20 September 2024) [22].

### 2.5. Detection of Genetic Diversity and Runs of Homozygosity (ROHs)

Nucleotide diversity (Pi) was calculated using VCFtools (v0.1.16) with a sliding window of 50 kb in length and a step size of 20 kb. Linkage disequilibrium (LD) was analyzed using PopLDdecay (v3.42), while the fixation index (FST) was computed using VCFtools (v0.1.16) to measure genetic differentiation across different breeds [23,24]. Runs of homozygosity (ROHs) were identified using PLINK (v1.90) and categorized into four groups based on their lengths: 0.5–1 Mb, 1–2 Mb, 2–3 Mb, and >3 Mb [25]. The ROH detection parameters included: (1) a window size of 50 SNPs (--homozyg-window-snp 50), (2) a minimum density requirement of one SNP per 50 kb (--homozyg-density 50), (3) a maximum of three heterozygotes allowed per window (--homozyg-window-het 3), and (4) a maximum of five missing calls allowed per window (--homozyg-window-missing 5).

### 2.6. Population Structure and Phylogenetic Analysis

The raw SNP data were initially filtered using the parameters --maf 0.05 (minor allele frequency) and --geno 0.2 (genotype missing rate per SNP), after which linkage disequilibrium (LD) pruning was performed using PLINK (v1.90) with the parameter --indep-pairwise 50 5 0.2. Principal Component Analysis (PCA) was conducted using the GCTA tool (v1.94.1), and the results were visualized using custom R scripts. To accurately identify the ancestral components of each breed, population structure analysis was performed using the ADMIXTURE software package (v1.3.0), with the number of ancestral populations (K) ranging from 2 to 6 [26]. The phylogenetic tree was constructed based on genetic distances derived from the distance matrix generated by PLINK (v1.90). For visualization and refinement, the phylogenetic tree was processed using MEGA (v11) and ITOL [27,28].

### 2.7. Detection of Gene Flow

Gene flow events between Yangba cattle and other cattle populations were inferred using the Fbranch module of the Dsuite software (v0.5 r53), with Jersey cattle serving as the out-group [29]. D-statistics were calculated using the Dquartets module, which heuristically explores all potential gene flow events within the ‘(((P1, P2), P3), P4)’ combination without requiring an out-group. To minimize false positives, only gene flow events with *p* < 0.01 and |*Z*| > 3 were considered significant. The ABBA-BABA method was employed to calculate gene flow values between Yangba cattle and other cattle populations, utilizing a sliding window approach (−w 20 kb) [29,30]. The top 5% of loci with the highest gene flow values were selected for gene annotation. These annotated genes were then subjected to Kyoto Encyclopedia of Genes and Genomes (KEGG) and Gene Ontology (GO) functional enrichment analyses.

### 2.8. Analysis of Selection Signals

To accurately analyze the genetic adaptation characteristics of Yangba cattle in their unique ecological environment, this study selected geographically and ecologically distinct Chinese taurine cattle and Chinese indicine cattle as control groups. The Chinese taurine cattle group (Tibetan cattle, Mongolian cattle, Yanbian cattle) represents types of cattle that have been domesticated over long periods in high-altitude cold and arid regions. The Chinese indicine cattle group (Leiqiong cattle, Wannan cattle, Jinjiang cattle, Dianzhong cattle, Wenshan cattle) includes breeds from subtropical to tropical humid climate zones, exhibiting adaptive features such as resistance to humidity, heat, and parasites. We employed four methods—FST (population differentiation index), XP-CLR (cross-population composite likelihood ratio test), XP-EHH (cross-population extended haplotype homozygosity), and nucleotide diversity (θπ)—to detect selection signals in Yangba cattle, aiming to screen for differential adaptive traits. FST analysis was conducted using VCFtools (v0.1.16) with a sliding window of 50 kb and a step size of 20 kb. Nucleotide diversity was estimated using VCFtools (v0.1.16) via a sliding window approach, with windows of 50 kb and a step size of 20 kb. SELSCAN was used to calculate XP-EHH statistics based on extended haplotypes for each population [31]. XP-CLR values between populations were calculated using the XPCLR tool (https://github.com/hardingnj/xpclr) (accessed on 8 November 2024). For all analyses, these thresholds (FST: top5%, θπ: top5%, XP-CLR: top5%, XP-EHH: top1%) were selected based on previous studies that have used similar methodologies in detecting selection signals [8,9,10,11]. Regions that passed the threshold criteria in multiple methods were considered as candidates for overlap filtering. This approach increases the reliability of the selected regions and ensures that the identified signals represent true selection events rather than random genetic drift. The identified candidate genes were subjected to functional analysis as well as GO and KEGG pathway enrichment analyses using the KOBAS 3.0 webserver [32].

## 3. Results

### 3.1. SNP Identification and Analysis of Genetic Diversity

**Figure 1:**
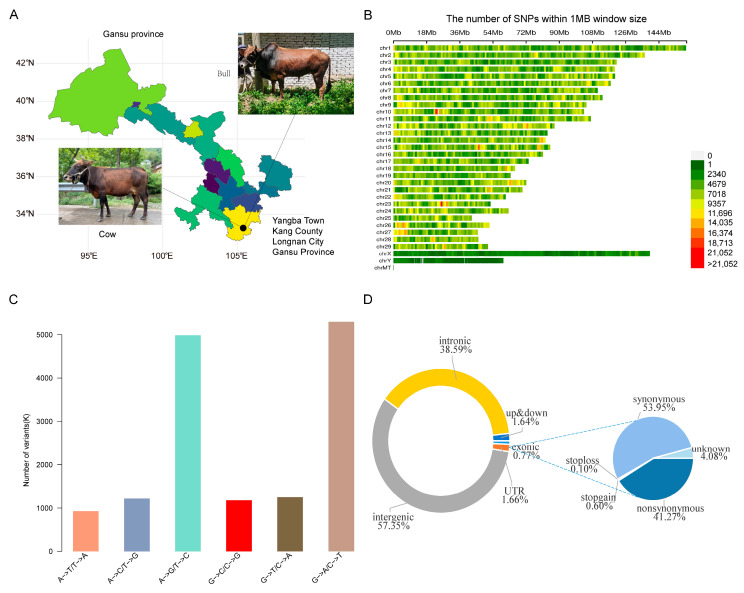
SNP Identification. (**A**) Geographical distribution of Yangba cattle: This map illustrates the geographical locations where Yangba cattle samples were collected. (**B**) Chromosomal distribution of SNPs: Each color in this figure represents the number of SNPs within a 1 Mb window across the chromosomes. (**C**) Base substitution patterns in Yangba cattle: This panel shows the different types of base substitutions (e.g., transitions and transversions) identified in the Yangba cattle genome. (**D**) Functional classification of genomic regions: This section classifies SNPs based on their genomic locations (e.g., coding regions, introns, regulatory elements).

Whole-genome resequencing of the 20 Yangba cattle (Figure 1A) generated 847.86 Gb of sequencing data after quality control, with an average sequencing depth of 14.54× and a mapping rate of 99.87% (Supplementary Table S3). After further filtering, we obtained 14,010,735 high-quality SNPs and 1,746,254 indels. A heatmap was created to illustrate the distribution of SNPs across chromosomes (Figure 1B), providing a clear visualization of SNP density. Mutation preferences and similarities among samples at the mutation level were analyzed based on the number and types of SNP mutations. The results indicated that the G→A and C→T mutations were the most prevalent types in the Yangba cattle population, whereas the A→T and T→A mutations were the least common (Figure 1C). The filtered SNPs were annotated using ANNOVAR, categorizing them based on genes, genomic regions, and functions. This analysis revealed that synonymous SNPs accounted for 53.95% of the exonic regions in Yangba cattle, while missense SNPs, stop gain SNPs, stop loss SNPs, and unknown SNPs comprised 41.27%, 0.6%, 0.1%, and 4.08%, respectively (Figure 1D).

**Figure 2:**
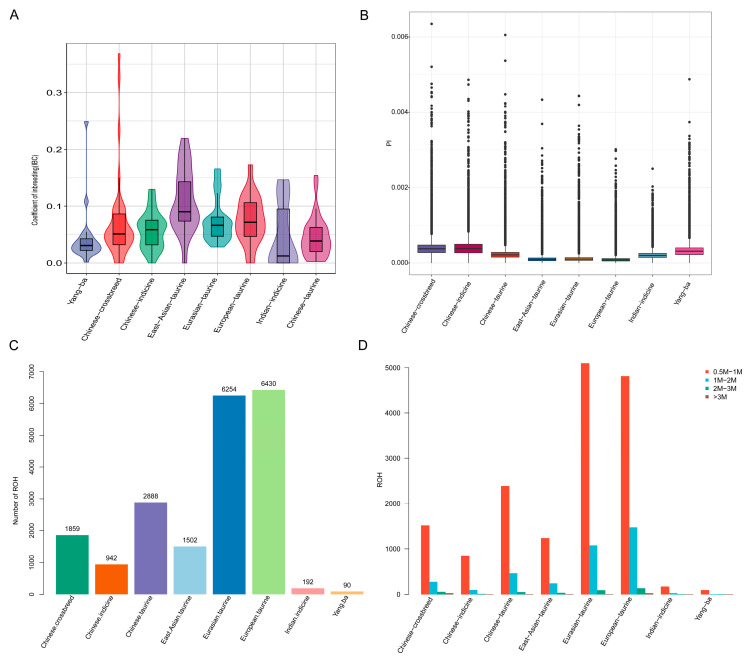
Analysis of genetic diversity. (**A**) Inbreeding coefficients across different populations, illustrating the degree of inbreeding within each group. (**B**) Nucleotide diversity (θπ) values for each population, reflecting the level of genetic variation. (**C**) Number of runs of homozygosity (ROHs) in different cattle populations, indicating the extent of homozygous segments. (**D**) Distribution of ROH segment lengths across different populations, showing the range and frequency of ROH sizes.

In accordance with previous research, the 202 samples in this study were categorized into seven distinct groups for comparative analyses: Indian indicine, East Asian taurine, Eurasian taurine, European taurine, Chinese taurine, Chinese hybrid cattle, and Chinese indicine (Supplementary Table S4) [33]. The inbreeding coefficients and nucleotide diversity within different populations were further evaluated. Results showed that East Asian, Eurasian, and European taurine cattle exhibited higher inbreeding coefficients, while Yangba cattle and local Chinese breeds displayed lower inbreeding coefficients. Indian indicine cattle had the lowest degree of inbreeding (Figure 2A). Nucleotide diversity analysis revealed that European breeds had significantly higher levels of polymorphism compared to local Chinese breeds (Figure 2B). Nucleotide diversity values reflect the base differences between any two individuals within a population and are closely related to selection intensity. The lower nucleotide diversity observed in Eurasian and European taurine cattle suggests they have undergone strong selection pressures during evolution. In contrast, local Chinese yellow cattle typically exhibit high genetic diversity. Notably, Yangba cattle in this study demonstrated comparatively high genetic diversity among domestic breeds, indicating their significant potential for genetic improvement.

Assessment of the number of runs of homozygosity (ROHs) across different populations revealed that Yangba cattle had the fewest ROHs, while European taurine cattle exhibited the highest number (Figure 2C). The ROHs were classified into four categories based on their lengths: 0.5–1 Mb, 1–2 Mb, 2–3 Mb, and >3 Mb. The findings indicated that ROHs were predominantly concentrated in the 0.5–1 Mb range. The longest homozygous segments were observed in European and Eurasian taurine cattle, whereas the shortest ROH segments were found in Yangba cattle (Figure 2D). The presence of continuous homozygosity suggests a closer genetic relationship among ancestors, typically indicating a higher degree of inbreeding. The significantly higher number of ROHs in European commercial breeds compared to Chinese cattle breeds can be attributed to extensive artificial selection, likely driven by intensive human intervention. This highlights the impact of selective breeding practices on the genetic structure of these populations.

### 3.2. Analysis of Population Structure

**Figure 3:**
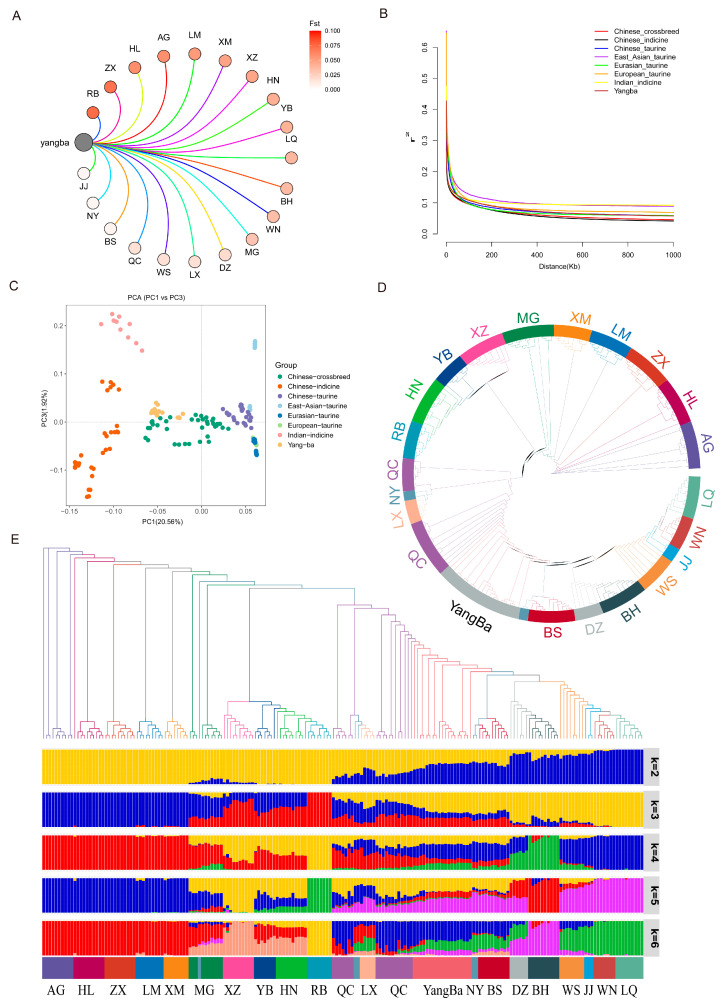
Analysis of population structure. (**A**) FST values between Yangba cattle and different cattle breeds, illustrating genetic differentiation. (**B**) LD decay across different populations, showing the rate of linkage disequilibrium decay. (**C**) PCA (principal component analysis) of different cattle populations, highlighting clustering patterns. (**D**) Phylogenetic tree of the different breeds of cattle, depicting evolutionary relationships. (**E**) Ancestry composition plot of the different breeds of cattle, indicating the proportional contributions of indicine and taurine lineages at various K values.

Analysis of FST values between Yangba cattle and other breeds revealed significant genetic differentiation between Yangba cattle and the Japanese, Jersey, and Hereford breeds, while showing higher genetic similarity with Jinjiang, Nanyang, and Bashan breeds (Figure 3A). Based on these findings, we hypothesize that Yangba cattle may represent a hybrid lineage of Chinese taurine and Chinese indicine cattle. The seven distinct populations were subjected to linkage disequilibrium (LD) analysis. The results showed that East Asian taurine cattle exhibited the highest levels of LD decay, whereas Chinese indicine cattle displayed the lowest levels. Notably, Yangba cattle showed relatively low LD decay (Figure 3B). The rate of LD decay reflects the extent to which alleles at different loci undergo recombination. East Asian taurine cattle demonstrated a slower rate of LD decay compared to other breeds, likely due to their ancestral relationships and lower genetic diversity. A higher degree of relatedness and reduced genetic variability decrease the recombination rate at loci, leading to broader LD ranges and slower LD decay. Conversely, the faster rate of LD decay observed in Yangba cattle indicates a higher rate of genetic recombination and an increased content of genetic information.

The principal component analysis (PCA) results revealed that Yangba cattle clustered closely with Chinese hybrid cattle (Figure 3C). To further ascertain the ancestral composition and lineage of Yangba cattle, we constructed a neighbor-joining phylogenetic tree and an ancestry contribution distribution plot based on genomic SNP data. The phylogenetic tree analysis demonstrated that most breeds formed distinct clusters, with Yangba cattle positioned between the Bashan and Qinchuan breeds (Figure 3D). The ancestry composition plot showed a clear separation into indicine and taurine lineages at K = 2, indicating that Yangba cattle possess both ancestries. At K = 3, the East Asian and Eurasian taurine lineages were distinguished, while at K = 4, the Chinese and Indian indicine cattle were differentiated (Figure 3E). Our analysis determined that the genetic ancestry of Yangba cattle is primarily composed of taurine and indicine lineages, with specific contributions as follows: 18% from Eurasian taurine, 26% from East Asian taurine, 39% from Chinese indicine, and 17% from Indian indicine lineages.

### 3.3. Analysis of Gene Introgression

**Figure 4:**
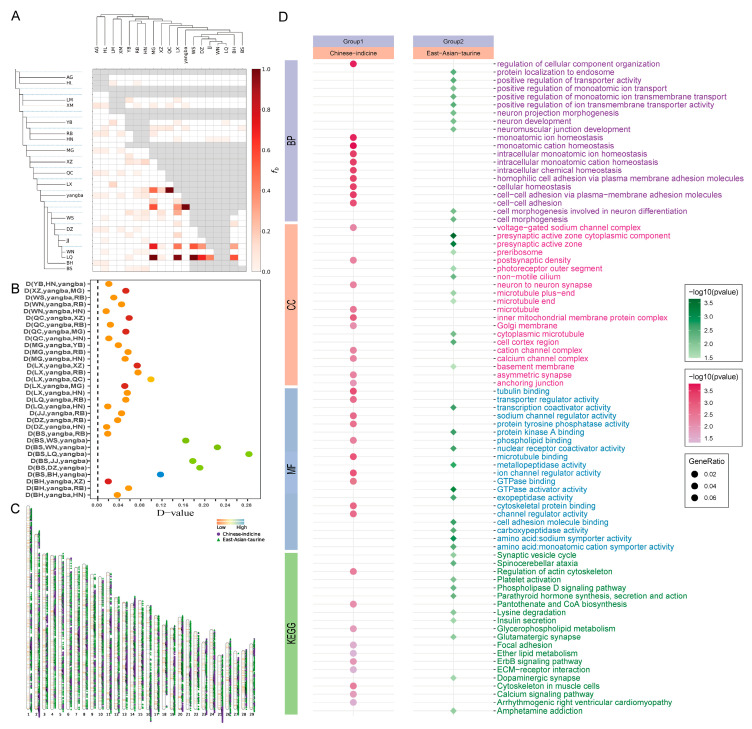
Analysis of gene introgression. (**A**) Heuristic analysis of gene exchange between Yangba cattle and various other cattle populations, highlighting patterns of gene flow. (**B**) Degree of gene exchange between Yangba cattle and other cattle populations, assessed using the F4 statistic. (**C**) Distribution map depicting gene introgression from Chinese indicine and East Asian taurine breeds into Yangba cattle across entire chromosomes, based on the top 5% gene flow values. (**D**) Results of KEGG and GO enrichment analyses of genes introgressed into Yangba cattle from Chinese indicine and East Asian taurine lineages, illustrating enriched pathways and biological functions.

Gene mixing events within the Yangba cattle population were inferred using heuristic analysis with the Fbranch module of Dsuite. The findings indicated gene flow between Yangba cattle and various East Asian taurine and indigenous Chinese indicine breeds (Figure 4A). To further assess the extent of gene introgression across 21 distinct breeds, we used the F4 statistic with Dsuite. The results demonstrated significant genetic exchange among Yangba, Chinese indicine, East Asian taurine, and Qinchuan cattle (Supplementary Table S5 and Figure 4B). The East Asian taurine and Chinese indicine populations, which showed significant gene exchange with Yangba cattle, were selected for subsequent analysis. The patterns of gene introgression from these lineages into Yangba cattle were calculated using the ABBA-BABAwindows.py script with a sliding window approach. The distribution of gene introgression was then plotted based on the top 5% gene flow values (Supplementary Table S6 and Figure 4C). Annotation of the genes in the top 5% of genomic regions revealed that 1279 and 904 genes had introgressed from East Asian taurine and Chinese indicine cattle, respectively, into Yangba cattle (Supplementary Table S7).

KEGG pathway enrichment analysis revealed that genes introgressed from East Asian taurine cattle are significantly enriched in pathways including amphetamine addiction, parathyroid hormone synthesis, secretion and action, phospholipase D signaling, platelet activation, lysine degradation, insulin secretion, and dopaminergic synapses. In contrast, genes introgressed from Chinese indicine cattle are significantly enriched in pathways related to the regulation of actin cytoskeleton, cytoskeleton organization in muscle cells, calcium signaling, focal adhesion, and ether lipid metabolism. GO enrichment analyses indicated that genes introgressed from East Asian taurine cattle predominantly participate in functions such as GTPase activator activity, positive regulation of transporter activity, amino acid: sodium symporter activity, and cell morphogenesis. On the other hand, genes introgressed from Chinese indicine cattle are primarily involved in processes like monoatomic cation homeostasis, regulation of cellular component organization, ion homeostasis, tubulin binding, and inner mitochondrial membrane protein complexes (Figure 4D).

### 3.4. Analysis of Selection Signals

#### 3.4.1. Adaptive Analysis of Indicine Lineage

**Figure 5:**
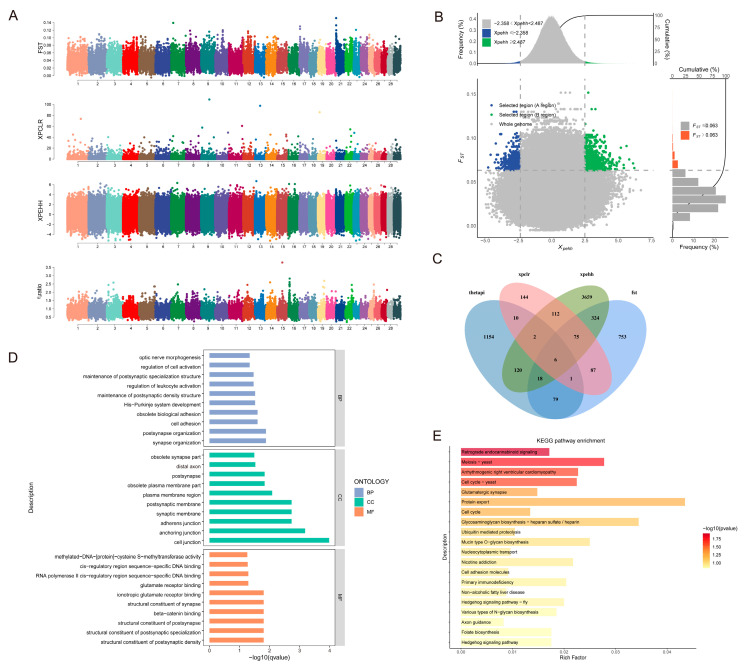
Adaptive analysis of indicine lineage. (**A**) Comparative analysis of genomic selection signals in Yangba and Chinese taurine cattle using four methods: FST, XP-CLR, XP-EHH, and θπ. This highlights the regions under selection in both populations. (**B**) Combined analysis of genomic selection signals in Yangba and Chinese taurine cattle using XP-EHH and FST, providing insights into shared and distinct selection pressures. (**C**) Venn diagram depicting the overlap of genes identified in Yangba and Chinese taurine cattle using different methods, illustrating common and unique candidate genes. (**D**) Results of GO enrichment analysis of candidate genes in Yangba cattle compared to those in Chinese taurine, showing enriched biological processes and molecular functions. (**E**) Results of KEGG pathway enrichment analysis of candidate genes in Yangba cattle compared to those in Chinese taurine, highlighting key metabolic and signaling pathways involved in adaptation and selection.

This section employed four analytical approaches (FST, XP-CLR, XP-EHH, and θπ) to examine adaptive divergence between the Yangba cattle lineage and Chinese taurine populations, specifically analyzing Tibetan cattle, Mongolian cattle, and Yanbian cattle as representative taurine groups. This analysis identified 1289, 485, 3591, and 1274 potential associated genes, respectively (Figure 5A,B). To ensure the reliability of our findings, we performed cross-validation on genes selected by three or more methods, ultimately identifying 109 candidate genes (Supplementary Table S8 and Figure 5C). GO enrichment analysis indicated that these candidate genes primarily participate in the regulation of organ growth, cell adhesion, heart growth, cardiac muscle tissue development, cell-cell junctions, and cell adhesion molecule binding (Figure 5D). KEGG pathway enrichment analysis revealed significant enrichment in pathways including riboflavin metabolism, insulin secretion, thyroid hormone signaling, adherens junctions, purine metabolism, phospholipase D signaling, renin secretion, and cortisol synthesis and secretion (Figure 5E). These pathways are involved in various physiological processes closely related to growth, metabolism, and hormonal regulation in cattle populations. Among the 109 overlapping candidate genes, GO enrichment analysis further highlighted significant biological processes such as cell adhesion, intracellular protein transport, and regulation of striated muscle tissue development. Key genes involved in these processes include *CTNNA3*, *TRAM1*, *PLCB1*, *FGFR2*, *AKAP6*, and *NR3C1*. These genes play crucial roles in cellular interactions, muscle development, and hormonal responses.

**Figure 6:**
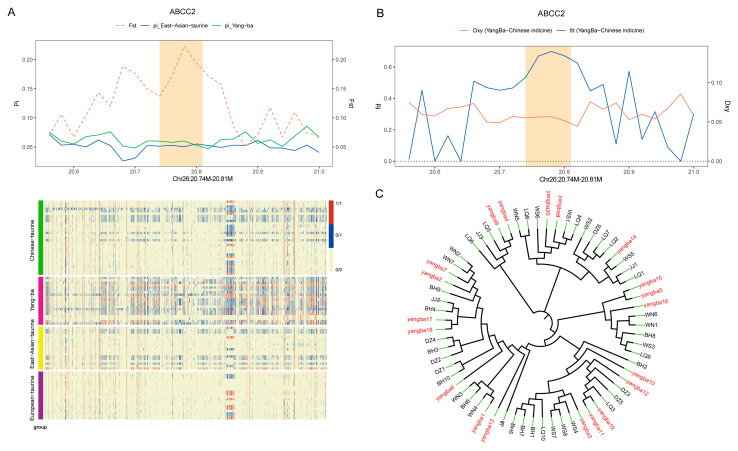
Characteristics of the ABCC2 region. (**A**) FST, θπ, and haplotype patterns in the ABCC2 region, illustrating genetic differentiation, nucleotide diversity, and linkage patterns. (**B**) Gene flow values (fd) and absolute divergence (Dxy) in the ABCC2 region, highlighting levels of genetic exchange and divergence between populations. (**C**) Phylogenetic tree of the ABCC2 region, depicting evolutionary relationships among different populations based on genetic variation in this locus.

Notably, in the significant GO term analysis of shared genes, *ABCC2* is involved in biological processes such as cellular response to endogenous stimuli, reproductive processes, response to peptide hormones, and cellular homeostasis. These processes highlight the crucial role of *ABCC2* in maintaining intracellular and extracellular stability and participating in hormonal signal transduction. Further analysis reveals that *ABCC2* is located on chromosome 26 and is primarily expressed in the liver and intestine. As a transport protein, *ABCC2* facilitates the transport of substances across cellular membranes, playing a vital role in detoxification and metabolic processes in these organs. To validate the function and selective signals of the *ABCC2* gene, we conducted haplotype analysis and constructed a phylogenetic tree in its region. The results indicated a high FST value for the *ABCC2* region, suggesting significant genetic differentiation among different populations. Additionally, the region showed high genetic diversity with substantial gene flow, indicating frequent genetic exchanges of *ABCC2* between populations and potential influences from natural selection (Figure 6A–C). These findings provide compelling evidence for the adaptive evolution of the *ABCC2* gene, further supporting its critical role in various biological processes.

#### 3.4.2. Adaptive Analysis of Taurine Lineage

**Figure 7:**
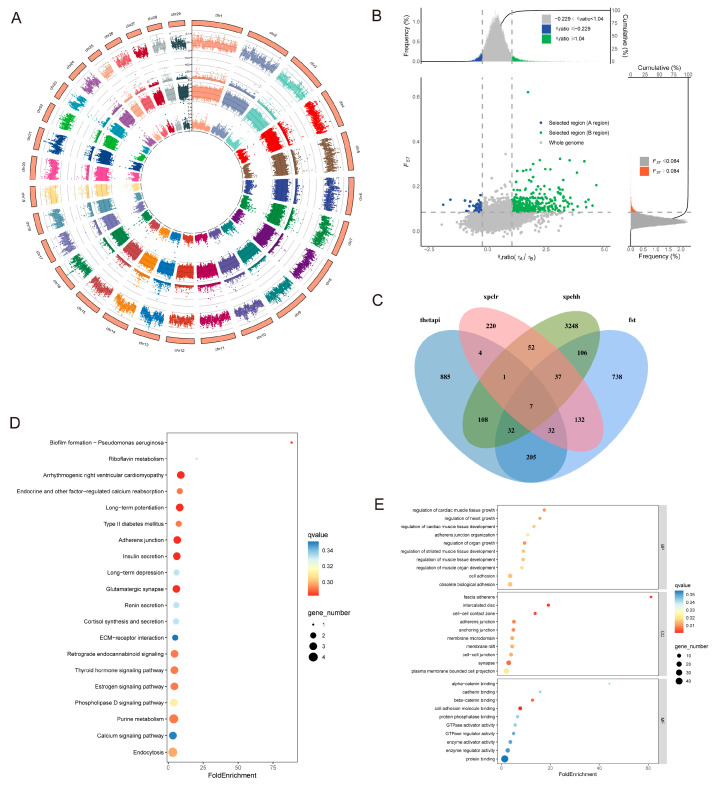
Adaptive analysis of taurine lineage. (**A**) Comparative analysis of genomic selection signals in Yangba and Chinese indicine cattle using four methods: FST, XP-CLR, XP-EHH, and θπ. This highlights the regions under selection in both populations. (**B**) Combined analysis of genomic selection signals in Yangba and Chinese indicine cattle using XP-EHH and FST, providing insights into shared and distinct selection pressures. (**C**) Venn diagram depicting the overlap of genes identified in Yangba and Chinese indicine cattle using different methods, illustrating common and unique candidate genes. (**D**) Results of KEGG pathway enrichment analysis of candidate genes in Yangba cattle compared to those in Chinese indicine, highlighting key metabolic and signaling pathways involved in adaptation and selection. (**E**) Results of GO enrichment analysis of candidate genes in Yangba cattle compared to those in Chinese indicine, showing enriched biological processes and molecular functions.

Focusing on taurine adaptation patterns, we utilized the same four methodologies (FST, XP-CLR, XP-EHH, and θπ) to investigate the Yangba cattle lineage in comparison with major Chinese indicine breeds, including Leiqiong cattle, Wannan cattle, Jinjiang cattle, Dianzhong cattle, and Wenshan cattle. This analysis identified 1343, 437, 4316, and 1390 potential associated genes, respectively (Figure 7A,B). To enhance the reliability of our results, we performed cross-validation on genes selected by at least three methods, ultimately identifying 102 candidate genes (Supplementary Table S9 and Figure 7C). KEGG pathway enrichment analysis indicated significant enrichment in pathways including protein export, glutamatergic synapse, retrograde endocannabinoid signaling, and arrhythmogenic right ventricular cardiomyopathy (Figure 7D). These pathways are involved in neural signal transmission, cardiac pathological processes, and molecular communication between cells. GO enrichment analysis revealed that these candidate genes primarily participate in functions such as methylated-DNA-[protein]-cysteine S-methyltransferase activity, cis-regulatory region sequence-specific DNA binding, ionotropic glutamate receptor binding, and beta-catenin binding (Figure 7E). Further GO enrichment analysis of the 102 overlapping candidate genes highlighted significant biological processes, including animal organ development, mitochondrial DNA metabolic processes, multicellular organism reproductive processes, heparin metabolic processes, and multicellular organism development. Key genes involved in these processes include *DNAJA3*, *PCDH9*, *CDC14A*, *EPHA5*, and *IMMP2L*. These genes likely play vital roles in regulating organ development, cellular metabolism, and developmental processes.

**Figure 8:**
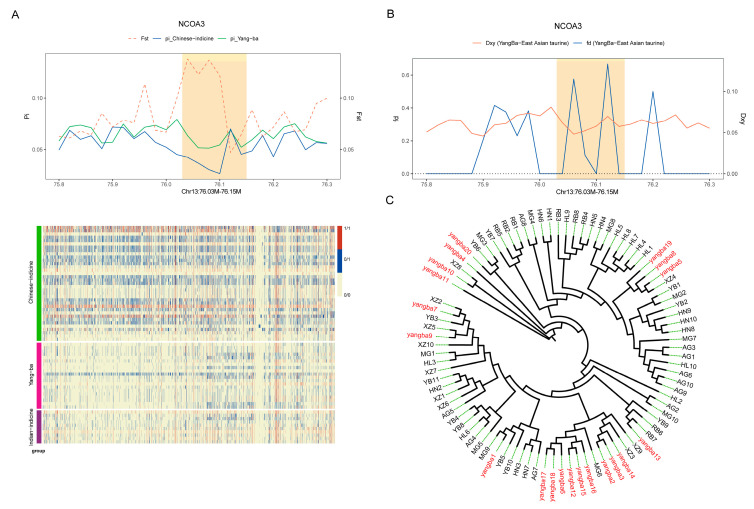
Characteristics of NCOA3 region. (**A**) FST, θπ, and haplotype patterns in the NCOA3 region, illustrating genetic differentiation, nucleotide diversity, and linkage patterns. (**B**) Gene flow values (fd) and absolute divergence (Dxy) in the NCOA3 region, highlighting levels of genetic exchange and divergence between populations. (**C**) Phylogenetic tree of the NCOA3 region, depicting evolutionary relationships among different populations based on genetic variation in this locus.

*NCOA3*, located on chromosome 13, encodes a coactivator protein pivotal in key cellular processes such as gene expression regulation, hormone signaling, and cell growth. In the significant GO term analysis of shared genes, *NCOA3* is classified as a crucial gene involved in the ‘cellular response to hormone stimulus’. To further validate this finding, we conducted haplotype and phylogenetic tree analyses of the region encompassing *NCOA3*. The results indicated that *NCOA3* exhibits high FST values, genetic diversity, and gene flow within its region, underscoring its significance in population genetics (Figure 8A–C). These analyses not only confirm the potential role of *NCOA3* in regulating hormonal responses but also reveal its genetic differences and adaptive significance across diverse populations.

## 4. Discussion

During the process of genetic evolution, the preference for mutation types is closely related to the selection pressures and external environments. Our study shows that the number of SNP mutations in Yangba cattle is relatively high, which may be one of the indirect factors leading to its population decline. Genetic diversity is a crucial foundation for biological evolution and species adaptation to environmental changes. Comparative analysis with multiple breeds revealed that the Yangba cattle population has fewer ROH (runs of homozygosity) and shorter ROH segments, along with a lower inbreeding coefficient and higher genetic diversity. High genetic diversity indicates that the Yangba cattle population contains a richer amount of polymorphic information, providing an advantage for adapting to various environmental selection pressures. The linkage disequilibrium (LD) analysis showed that the LD decay distance in Yangba cattle is relatively short, indicating a faster decay rate. This further suggests that the Yangba cattle population has a wide range of gene variation and a higher rate of genetic recombination. Compared to other domestic and international breeds, Yangba cattle exhibit higher genetic diversity, indicating that they may harbor unique genetic resources.

Although Yangba cattle are located at the border of the Central Plains, their bloodline origins and genetic background have not been clearly defined. Our analysis identified a hybrid ancestry in Yangba cattle, comprising both taurine and indicine lineages. Specifically, 18% of the genetic contribution comes from Eurasian taurine, 26% from East Asian taurine, 39% from Chinese indicine, and 17% from Indian indicine. This genetic composition is similar to that of other breeds in the nearby Sichuan Basin region, such as Bashan cattle and Sanjiang cattle [34,35]. Further introgression analysis revealed that genes introgressed from East Asian taurine are enriched in pathways related to metabolism, signal transduction, cellular morphology changes, and neural functions. In contrast, genes introgressed from Chinese indicine are significantly enriched in pathways involving the cytoskeleton, calcium signaling, muscle cell function, and focal adhesion. This differential functional divergence of introgressed genes may be associated with the complex selection pressures in the Qinba mountainous region—metabolic regulation and neural development adaptations to meet the dietary needs imposed by the diverse vegetation in subtropical mountain environments, while muscle function enhancement responds to the physical demands of navigating steep terrain. Additionally, our introgression analysis indicated significant gene flow between Yangba cattle and Qinchuan cattle, likely due to local population mixing.

Through comparative genomic selection signal analysis with Chinese taurine cattle, we identified several genes in the Yangba cattle population, including *CTNNA3*, *ABCC2*, *PLCB1*, *FGFR2*, *AKAP6*, and *NR3C1*, showing signs of positive selection. Specifically, functional enrichment results suggest that *CTNNA3* and *ABCC2* may be associated with immune adaptability, *PLCB1* with heat tolerance, and *FGFR2*, *AKAP6*, and *NR3C1* with muscle development. These findings align with the unique advantages of Yangba cattle in adapting to the local humid and hot environment and their growth performance. *CTNNA3* is a crucial protein involved in cell adhesion and signal transduction, regulating cell migration and proliferation. Previous studies have shown that *CTNNA3* is linked to growth traits and disease resistance in various animals; for instance, in sheep, *CTNNA3* has been associated with growth traits and susceptibility to diseases [36,37]. *ABCC2* is a transporter protein primarily involved in the transport of glutathione, thereby regulating amino acid metabolism and cellular ferroptosis, playing a significant role in immune adaptability [38]. *PLCB1* plays an important role in signal transduction, and studies have indicated its close association with heat tolerance. Aboul-Naga and Xie found that the *PLCB1* gene exhibited adaptive characteristics under heat stress conditions in rabbits and sheep, respectively [39,40]. Our study also detected a trend of positive selection for the *PLCB1* gene in Yangba cattle, which may contribute to their survival and reproduction in high-temperature environments. *FGFR2*, *AKAP6*, and *NR3C1* are all involved in muscle development and metabolic processes. *FGFR2* regulates myogenesis in skeletal muscle stem cells [41], *AKAP6* plays a crucial role in myoblast differentiation, myotube formation, and muscle regeneration [42], and *NR3C1* is key in metabolic processes affecting muscle size, mass, and function [43]. As mentioned, these genes predominantly act on immune system activation and heat adaptation, making them important candidate genes for tropical adaptation. Therefore, we infer that the positive selection of these genes likely aids Yangba cattle in adapting to the local humid and hot environment, enhancing their survival competitiveness.

Compared to Chinese indicine cattle, several candidate genes under significant positive selection were identified in the taurine lineage of Yangba cattle. These genes can be categorized into growth-related genes (*DNAJA3*, *PCDH9*, *EPHA5*, *NCOA3*) and reproduction-related genes (*CDC14A*, *IMMP2L*). The adaptive evolutionary characteristics of these genes provide insights into the genetic mechanisms underlying important traits in Yangba cattle. Among the growth-related genes, *DNAJA3* has been found to have dual biological functions. This gene not only maintains homeostasis by regulating B cell development and immune responses but also potentially influences muscle development through its chaperone activity in protein folding [44]. As a member of the protocadherin family, *PCDH9* exhibits pleiotropic functions: in addition to participating in cell adhesion and signal transduction during neural development [45], a GWAS study in Chinese Holstein cattle confirmed that single nucleotide polymorphisms in *PCDH9* are significantly associated with birth weight [46]. The regulatory role of *EPHA5* in body size development is supported by evidence from multiple species, including genomic scans in Haimen goats [47] and homozygosity analysis in Hu sheep [48]. *NCOA3*, as a nuclear receptor coactivator, integrates hormone signaling pathways to regulate the balance between cell proliferation and apoptosis; loss of function can lead to cell cycle arrest and increased apoptosis [49]. Recent studies have also found that *NCOA3* plays a role in DNA damage repair mechanisms [50]. Regarding reproduction-related genes, *CDC14A*’s critical role in male reproduction has been validated through knockout experiments. Studies using mouse models have shown that the absence of *CDC14A* leads to abnormalities in meiosis, sperm morphology defects, and reduced motility, ultimately resulting in infertility [51]. *IMMP2L*, an inner mitochondrial membrane processing enzyme, has been linked to reproductive performance in Qianhua meat Merino sheep and Iranian camels [52,53], suggesting that this gene may also be associated with the reproductive performance of Yangba cattle.

In the analysis of gene flow and genomic selection signals, we identified multiple key genes, including *ABCC2*, *CTNNA3*, *CSMD3*, *CADM2*, *NCOA3*, *PCDH9*, *NCAM2*, and *VPS13B*. The functional enrichment results of these genes are mainly related to cellular function regulation, disease resistance, and muscle development. We speculate that Yangba cattle may have acquired these biologically significant genes from different ancestral sources during evolution, forming notable local adaptive traits over time, thereby enhancing their ability to adapt to environmental changes. However, our SNP-based analysis did not cover the potential contributions of structural variations and epigenetic regulation to the phenotype, which might underestimate the impact of genomic complexity on adaptability. Given the limited number of existing Yangba cattle, this study conducted whole-genome resequencing on 20 representative samples. These samples were selected from 12 locations in Longnan City’s Kang County, including Baidang Town, Lianghe Town, Yangba Town, Tongqian Town, and Sanhe Town, with a minimum spacing of 10 km between each sampling location. Nevertheless, due to limited geographic coverage, unique genetic features of marginal subpopulations may have been overlooked, and the detection power for low-frequency alleles (MAF < 0.05) might be insufficient. In the future, our research will deepen the biological findings of this study by expanding the sample collection scope, supplementing phenotypic data, and validating the functions of candidate genes.

## 5. Conclusions

The comparative genomic analysis in this study provides new insights into the ancestry and genetic diversity of Yangba cattle, offering crucial theoretical support for the conservation and improvement of this breed. Simultaneously, through this research, the genetic information and diversity of Yangba cattle have been explored and enhanced, increasing the diversity and value of existing genetic resources. Moreover, we identified several candidate genes related to important economic traits (such as muscle development and reproduction) and adaptability (resistance) at the genomic level. These findings further provide significant references for the utilization and protection of endangered Yangba cattle resources and the implementation of cattle breeding programs.

## Data Availability

The data that support the findings of this study are available from the corresponding author upon reasonable request.

## Supplementary Materials

The following supporting information can be downloaded at: https://www.mdpi.com/article/10.3390/ani15071065/s1, Table S1: Information on the serial numbers of 182 samples; Table S2: Genomic statistics for 182 samples; Table S3: Sequencing statistics for the Yangba cattle; Table S4: Grouping of samples; Table S5: Statistics of gene infiltration; Table S6: Window statistics of East Asian taurine and Chinese indicine infiltration into Yangba cattle; Table S7: Genetic statistics of East Asian taurine and Chinese indicine infiltration into Yangba cattle; Table S8: Positive selection gene statistics of the indicine lineage in Yangba cattle; Table S9: Positive selection gene statistics of the taurine lineage in Yangba cattle.

## Abbreviations

The following abbreviations are used in this manuscript:

SNP	Single nucleotide polymorphism
MAF	Minor allele frequency
WGS	Whole-genome sequencing
θπ	Nucleotide diversity
XP-EHH	Cross-population extended haplotype homozygosity
GO	Gene ontology
KEGG	Kyoto Encyclopedia of Genes and Genomes
NJ	Neighbor-joining
PCA	Principal component analysis
ROH	Runs of homozygosity
LD	Linkage disequilibrium
Fst	Fixation index
fd	Gene flow values
Dxy	Absolute divergence
AG	Angus cattle
HL	Hereford cattle
ZX	Jersey cattle
LM	Limousin cattle
XM	Simmental cattle
MG	Mongolian cattle
XZ	Tibetan cattle
YB	Yanbian cattle
HN	Korean cattle
RB	Japanese cattle
QC	Qinchuan cattle
LX	Luxi cattle
Yang Ba	Yangba cattle
NY	Nanyang cattle
BS	Bashan cattle
DZ	Dianzhong cattle
BH	Indian cattle
WS	Wenshan cattle
JJ	Jinjiang cattle
WN	Wannan cattle
LQ	Leiqiong cattle
